# Pathogen sharing in the digital age: The unfinished agenda of the WHO pandemic agreement

**DOI:** 10.1002/ctm2.70473

**Published:** 2025-09-05

**Authors:** WooJung Jon

**Affiliations:** ^1^ Korea Advanced Institute of Science and Technology (KAIST) Daejeon South Korea

## 1 THE PABS SYSTEM: INNOVATION AND CONTROVERSY

The world cheered on May 20, 2025, when the World Health Assembly adopted the World Health Organization (WHO) Pandemic Agreement – a landmark global treaty forged after three years of intense negotiations – hailed as a triumph of multilateralism.^1^ This accord, the first‐ever treaty focused on pandemic prevention, preparedness, and response, embodies a collective resolve to avert a repeat of the devastation caused by COVID‐19. However, this celebratory moment is not the end of the story; rather, it is the beginning of a far more challenging chapter.

At the heart of the Pandemic Agreement is its most innovative and contentious element: a Pathogen Access and Benefit‐Sharing (PABS) system (Article 12). PABS was envisioned as the Agreement's grand bargain to correct the inequities observed during COVID‐19, when pathogens were often detected in the Global South but vaccines and therapeutics were hoarded by the Global North.^2^ Under the PABS framework, countries are required to promptly share pathogen samples and genetic sequence data; in return, pharmaceutical manufacturers using that data must share the benefits. The key commitment is for companies to provide 20% of their real‐time pandemic production to the WHO for distribution based on public health need (Article 12.6(a) ). At least half of this (10% of total production) would be donated outright, and the rest would be offered at affordable prices. This mechanism aims to replace the chaotic, market‐driven scramble for life‐saving tools with a predictable, needs‐based system that ensures more equitable access.

Yet the technical and political complexities of PABS proved too difficult to resolve during the main treaty negotiations. To avoid a collapse of the talks, member states took a high‐stakes step: they adopted the core agreement text but deferred all operational details of PABS to a separate, legally binding annex.^3^ Pursuant to Article 31.2, the Pandemic Agreement cannot be opened for signature or ratification until this PABS annex is successfully negotiated and adopted.^1^ This task now falls to a new Intergovernmental Working Group (IGWG), which convened in July 2025 with a mandate to complete the annex by the 2026 World Health Assembly.^4^

## 2 DIGITAL SEQUENCE INFORMATION: THE GOVERNANCE CHALLENGE

A major complication for PABS is the role of digital sequence information (DSI) – essentially the genetic code of pathogens stored as data. DSI allows researchers and companies to work with a pathogen's genome without needing the physical virus or bacteria in hand. For example, the first COVID‐19 vaccines (like the mRNA vaccines) were designed using the digitally shared SARS‐CoV‐2 genome sequence, without any laboratory exchanging live virus samples.^5^ This ability to ‘dematerialize’ pathogens into data means that a company can obtain the genetic sequence of a dangerous pathogen from a public database and use it to develop products – effectively bypassing traditional pathogen‐sharing arrangements that focus on physical specimens.

Even though Article 12 covers pathogen ‘sequence information’, practical enforcement is challenging – a user might claim to have obtained a sequence from a public database and thus seek to avoid benefit‐sharing. The drafters of the Pandemic Agreement tried to address this by explicitly including ‘sequence information’ alongside physical materials in the scope of PABS in Article 12. In theory, this means that even the use of a pathogen's genetic sequence (not just the virus vial itself) should invoke the duty to share benefits. In practice, however, enforcing such obligations for digital data is tricky.^6^ Pathogen genetic sequences are often uploaded to open databases (e.g., GISAID or GenBank) for the global scientific community. Unless a traceability system is put in place – one that can tag and monitor which sequences are part of the PABS system – manufacturers could claim they obtained the sequence from a non‐PABS source and argue they owe no benefits.^7^ Crafting rules for tracking the use of DSI, ensuring open access for researchers, yet preventing free riders who exploit data without sharing back, is a delicate balance that the PABS annex negotiations will need to tackle.**⁷** Simply put, DSI empowers science to outrun governance: pathogens can now spread digitally as information just as fast as they spread biologically, and any effective benefit‐sharing system must account for that reality.

## 3 JURISDICTIONAL OVERLAP WITH THE CBD FRAMEWORK

The emergence of PABS has collided with a parallel global framework under the United Nations Convention on Biological Diversity (CBD)^8^ dealing with digital sequence information. At the 16th meeting of the Conference of the Parties to the CBD (COP‐16), held in Cali, Colombia, from October 21 to November 1, 2024, the Parties adopted Decision 16/2 on digital sequence information on genetic resources, operationalising a multilateral benefit‐sharing mechanism and establishing the ‘Cali Fund for the Fair and Equitable Sharing of Benefits from the Use of Digital Sequence Information on Genetic Resources’.^9^ Paragraph 3 of the Annex to Decision 16/2 provides that eligible commercial users should contribute to the Fund at indicative rates of 1% of annual profits or 0.1% of annual revenue.^9^ CBD Parties are ‘invited’ to implement national incentives or measures to promote such contributions (Decision 16/2, Annex, para 13).^9^ By contrast, the WHO's PABS system will be a specialised, binding mechanism focused solely on pathogens that mandates in‐kind contributions (20% of production) rather than cash.

**FIGURE 1:**
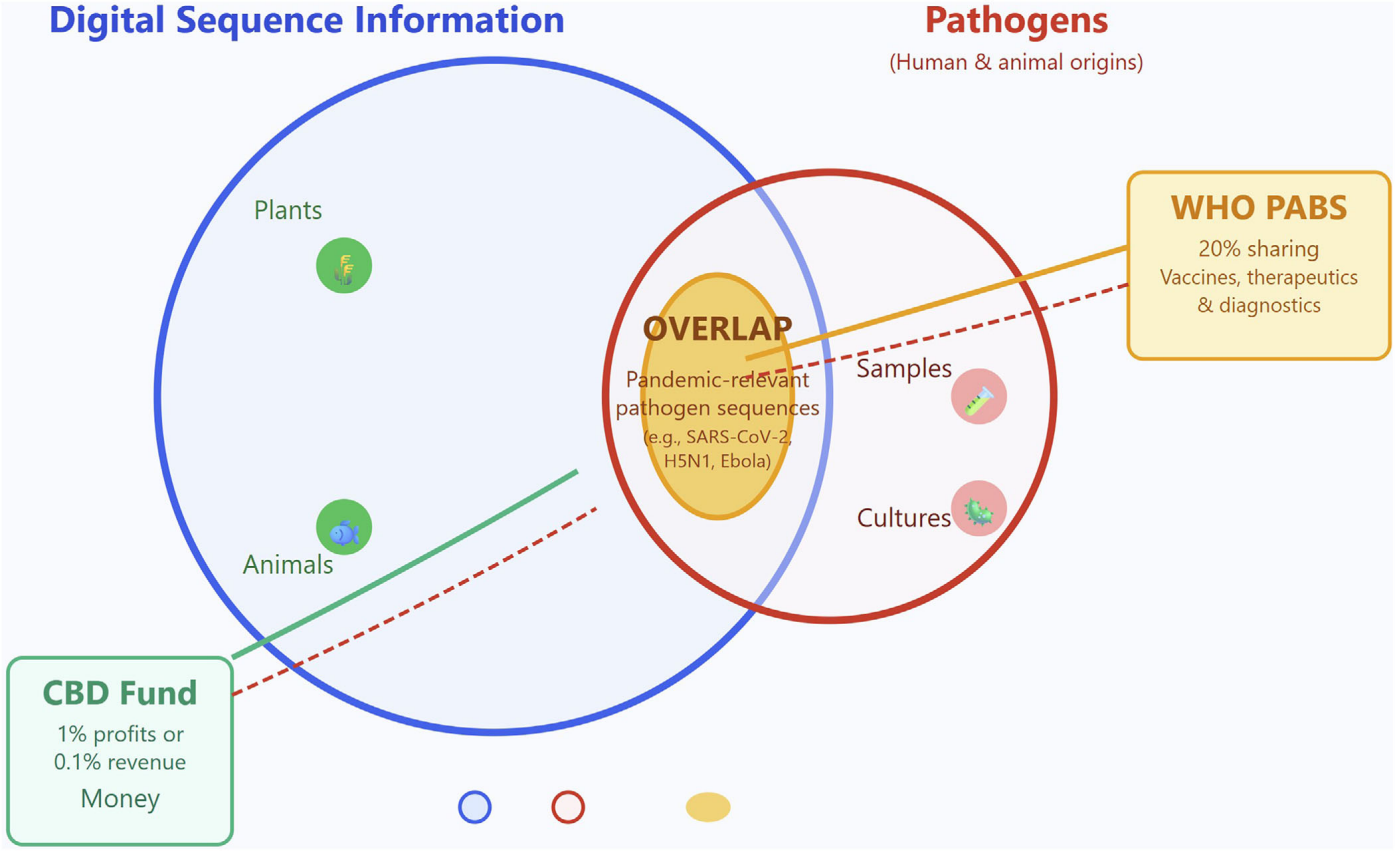
Jurisdictional overlap of CBD DSI mechanism and WHO PABS.

The conflict arises because the CBD's broad system explicitly includes pathogens, which are the exclusive focus of the PABS system. This jurisdictional overlap, where the CBD's general framework covers all genetic resources and the WHO's specialised system focuses only on pathogens, creates a potential legal conflict in which a company could face contradictory demands from both frameworks for the same activity (see Figure 1).

The diagram illustrates the conflict between the CBD's general framework covering all DSI and the WHO's specialised PABS system for pathogens. The overlap represents the area of legal contention, where double benefit‐sharing – monetary for the CBD and in‐kind for the WHO – might be demanded for the use of DSI from pathogens with pandemic potential.

## 4 LEGAL SOLUTIONS AND POLITICAL REALITIES

The intended legal solution to this jurisdictional overlap is the principle of *lex specialis derogat legi generali* (a specific law overrides a general one). The Pandemic Agreement itself lays the groundwork for this solution. Article 12.4 explicitly references Article 4.4 of the Nagoya Protocol, stating that the PABS system ‘shall be consistent with, and not run counter to, the objectives of the Convention on Biological Diversity and the Nagoya Protocol’. This clause is a deliberate legal manoeuvre to position the PABS system as a ‘specialized international access and benefit‐sharing instrument’ under the Nagoya Protocol's framework.^10^ Once recognized as a ‘specialized instrument’ under the Nagoya Protocol, PABS would take priority over the CBD’s general DSI mechanism for benefit‑sharing related to pathogens with pandemic potential. However, until such recognition is formally granted by the CBD's own Conference of the Parties, the legal uncertainty and potential for conflict remain, discouraging the very innovation needed to fight pandemics.^7^

While the legal principle of *lex specialis* offers a pathway, transforming this doctrine into operational reality remains a political challenge rather than a legal certainty. To address this, the WHO should prioritise securing formal recognition of PABS as a ‘specialized instrument’ under the Nagoya Protocol,^10^ and the CBD should establish clear exemptions for pandemic‐related pathogen DSI, thereby preventing jurisdictional overlap that could impede life‐saving innovation.

## AUTHOR CONTRIBUTIONS

WooJung Jon (WJJ) is the sole author. WJJ conceived the study; designed the methodology; conducted the legal and policy analysis; curated sources; drafted, revised and approved the final manuscript; and is the guarantor who accepts full responsibility for the overall integrity of the work.

## CONFLICT OF INTEREST STATEMENT

The author declares no competing interests.

## ETHICS STATEMENT

This article reports a desk‑based legal–policy analysis using publicly available sources only. It did not involve human participants, patient data, or animal subjects. Accordingly, institutional ethical approval and informed consent were not required.
